# Outplanting technique, host genotype, and site affect the initial success of outplanted *Acropora cervicornis*

**DOI:** 10.7717/peerj.4433

**Published:** 2018-02-28

**Authors:** Elizabeth A. Goergen, David S. Gilliam

**Affiliations:** Halmos College of Natural Sciences and Oceanography, Nova Southeastern University, Dania Beach, FL, USA

**Keywords:** Coral nursery, Florida, Productivity, Coral Point Count, Propagation, Restoration

## Abstract

*Acropora cervicornis* is the most widely used coral species for reef restoration in the greater Caribbean. However, outplanting methodologies (e.g., colony density, size, host genotype, and attachment technique) vary greatly, and to date have not been evaluated for optimality across multiple sites. Two experiments were completed during this study, the first evaluated the effects of attachment technique, colony size, and genotype by outplanting 405 *A. cervicornis* colonies, from ten genotypes, four size classes, and three attachment techniques (epoxy, nail and cable tie, or puck) across three sites. Colony survival, health condition, tissue productivity, and growth were assessed across one year for this experiment. The second experiment assessed the effect of colony density by outplanting colonies in plots of one, four, or 25 corals per 4 m^2^ across four separate sites. Plot survival and condition were evaluated across two years for this experiment in order to better capture the effect of increasing cover. Colonies attached with a nail and cable tie resulted in the highest survival regardless of colony size. Small corals had the lowest survival, but the greatest productivity. The majority of colony loss was attributed to missing colonies and was highest for pucks and small epoxied colonies. Disease and predation were observed at all sites, but did not affect all genotypes, however due to the overall low prevalence of either condition there were no significant differences found in any comparison. Low density plots had significantly higher survival and significantly lower prevalence of disease, predation, and missing colonies than high density plots. These results indicate that to increase initial outplant success, colonies of many genotypes should be outplanted to multiple sites using a nail and cable tie, in low densities, and with colonies over 15 cm total linear extension.

## Introduction

The compounding effects of human population growth, coastal construction, and climate change have caused damage to coral reef ecosystems worldwide (Schopmeyer et al., 2012; Bégin et al., 2016; Bright et al., 2016; Hume et al., 2016; Towle, Baker & Langdon, 2016). Historically, the Caribbean staghorn coral, *Acropora cervicornis*, was one of the most important corals in terms of contributing to habitat complexity and reef framework, playing a vital role in the reef community (Goreau, 1959; Goreau & Goreau, 1973; Adey & Burke, 1977; Neigell & Avise, 1983). The mainly monotypic stands of *A. cervicornis*, also referred to as thickets, fields, stands or patches, lined the fore and back reefs, spur tops, and octocoral dominated reefs of many Caribbean, Florida, and Gulf of Mexico reefs (Davis, 1982; Bruckner, 2002; Acropora Biological Review Team, 2005). Its fast growth rate and natural ability to fragment allows it to spread across habitats quickly forming dense patch-like structures providing habitat to a multitude of vertebrate and invertebrate species.

More recently (since the 1980s) populations within the Greater Caribbean have become regionally isolated, existing most commonly as individual colonies or much smaller patches separated by several kilometers or more. The major decrease in the species seen throughout the Caribbean in the 1970s and 1980s was caused by a white band disease outbreak (Gladfelter, 1982; Bythell et al., 1989; Bythell, Gladfelter & Bythell, 1993; Aronson & Precht, 2001; Acropora Biological Review Team, 2005). Since this dramatic decline, recovery has been limited with few known high cover populations remaining throughout the species range (Vargas-Ángel, Thomas & Hoke, 2003; Keck et al., 2005; Grober-Dunsmore, Bonito & Frazer, 2006; Lirman et al., 2010; Walker et al., 2012; D’Antonio, Gilliam & Walker, 2016). With the loss of these three-dimensional structures comes the loss of an unprecedented amount of habitat. In 2006, *A. cervicornis* was listed as threatened under the US Endangered Species Act (National Marine Fisheries Service, 2006) and in 2008, listed as critically endangered on the World Conservation Union (IUCN) red list (Aronson et al., 2008). While controlling stressors like human population growth, coastal construction, and climate change is difficult, it is as challenging to perform coral reef restoration in the face of these stressors. However, together with effective and active management plans we can use coral reef population enhancement techniques to attempt to increase resilience of the remaining populations.

Restoration activities specifically for *A. cervicornis* began in 2001 (Bowden-Kerby, 2001) and have since increased exponentially (Johnson et al., 2011). Young, Schopmeyer & Lirman (2012) reported over 60 programs working on *Acropora* spp. restoration in the Caribbean. Most of these programs are successfully increasing the abundance of *A. cervicornis* on numerous reefs and are now collectively outplanting tens of thousands of corals a year (Schopmeyer et al., 2017). As mass outplanting becomes more common the best techniques to ensure initial colony survival and growth need to be determined. Outplant designs should incorporate experimentally derived best practices for appropriate colony size, density, attachment technique, site, and host and symbiont genotypes (Griffin et al., 2012; Hollarsmith, Griffin & Moore, 2012; Lirman et al., 2014; Mercado-Molina, Ruiz-Diaz & Sabat, 2015). In this study, we evaluated the effect of host genotype, density, outplant size, and attachment techniques across multiple sites on initial success (within one to two years) of outplanting. It is important that colonies survive and grow large enough during the first year following outplanting so that they can contribute to natural populations through sexual reproduction and fragmentation. It is also important to understand differences amongst genotypes and their growth, survival, and health under the same environmental conditions (same outplant site), as these results could inform restoration practices and improve success. For example, genetic diversity increases the likelihood of successful sexual reproduction, and outplanting slower growing genotypes at larger sizes would allow them to contribute to sexual reproduction more quickly. Therefore, success herein is defined by initial colony survival in the location in which they were outplanted similar to that observed in other population enhancement programs or in the wild (>50%), colonies are exhibiting growth and productivity (increasing abundance and complexity on the reef) and relatively low prevalence of disease and predation.

## Methods

### Size and attachment technique

**Figure 1:**
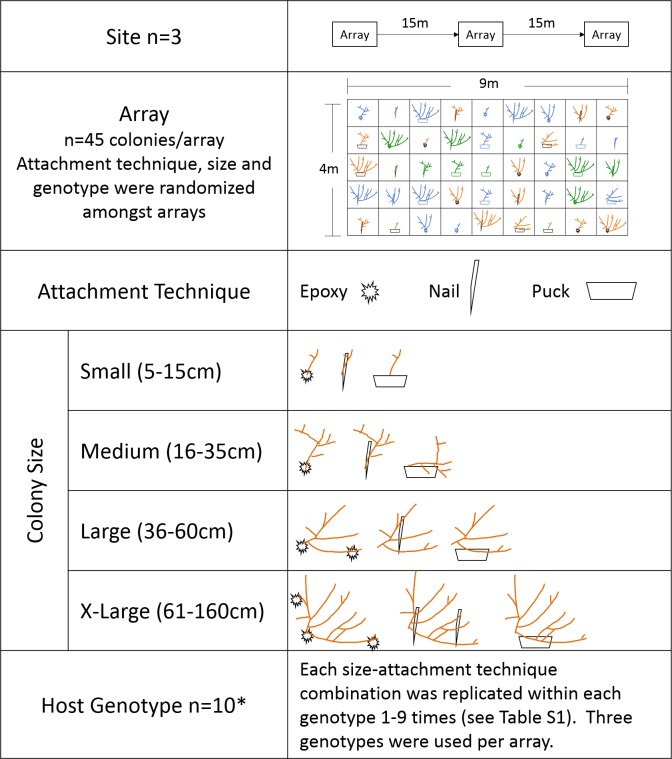
Schematic of experimental design of the size and attachment technique experiment. Different colors in the array diagram represent genotypes. *ten genotypes were used, however because of the low number of replicates for Genotype 2 it was removed from genotype analyses.

**Figure 2:**
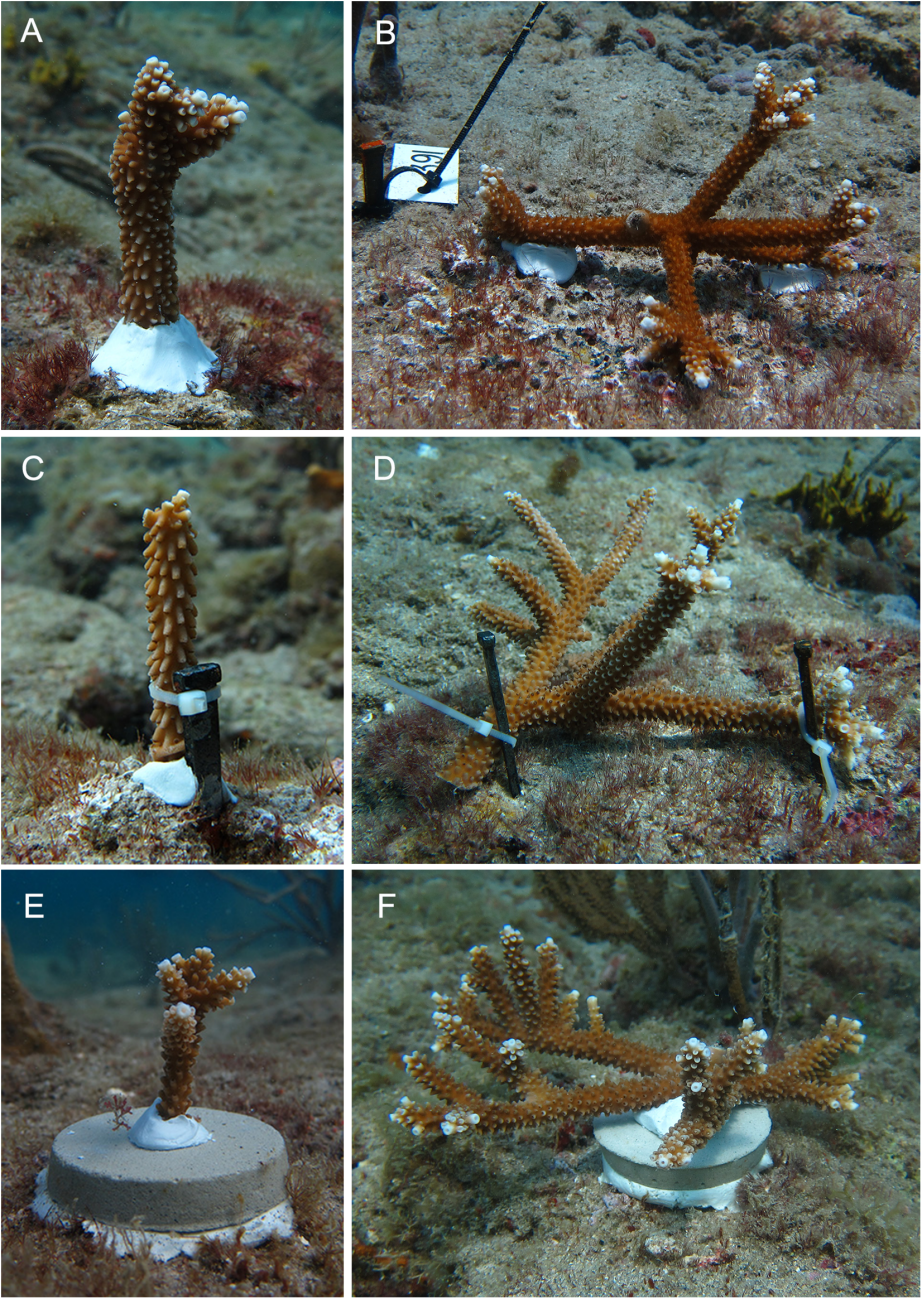
Outplanted *Acropora cervicornis* colonies using three attachment techniques: two-part epoxy (A, B), masonry nail and cable tie (C, D) or cement puck (E, F) and four colony size classes: small (5–15 cm), medium (16–35 cm), large (36–60 cm), and x-large (>60 cm), pictured here are small, vertically outplanted (A, C, E) and x-large, horizontally outplanted colonies (B, D, F). To better ensure colony stabilization the small size class was outplanted vertically and the larger size classes horizontally.

Corals were outplanted March 2015 to three sites on the nearshore ridge complex of Broward County, Florida at depths between 4 and 5 m. At each site, corals were outplanted to three arrays using ten genotypes, three attachment techniques: (1) two-part epoxy (“epoxy”), (2) masonry nail and cable tie (“nail”) or (3) cement puck (“puck”), and four size classes: (1) small (5–15 cm total linear extension (TLE)), (2) medium (16–35 cm TLE), (3) large (36–60 cm TLE), and (4) x-large (61–160 cm TLE). Coral host genotype was previously determined by Baums et al. (2010) using microsatellite markers. Forty-five colonies were outplanted to each array with genotype, colony size, and attachment technique randomly assigned within each array at each site (Fig. 1). Small colonies for all attachment techniques and medium nail colonies were outplanted upright, whereas medium puck, medium epoxy, and all large and x-large colonies were transplanted horizontally to ensure colony stability (Fig. 2). Medium and large/x-large epoxy colonies were attached with two and three epoxy points, respectively. Each size class/attachment technique combination was replicated a minimum of 27 times within the three sites for a total of 405 corals (Table S1).

Monitoring occurred at 1, 4, 8, and 12 months post-outplanting. Individual colony survival (alive, dead, or missing), percent tissue mortality, and prevalence of conditions (predation, disease, and bleaching) were recorded. Colonies were considered alive if they were found in the location where they were outplanted and any live tissue was still present. The cumulative prevalence of each condition was calculated by adding the number of colonies affected by each condition during the year divided by the sum of susceptible colonies (colonies with live tissue) during the same period.

Colony growth and productivity analysis was completed using images of each coral taken from the same direction, with a scaling object for calibration, taken upon outplanting and at one year post-outplanting. TLE per colony (sum of all branch lengths and central axis) was determined using the tracing feature in Coral Point Count with Excel extensions 4.1 (CPCe)© (Kohler & Gill, 2006) (Fig. S1). Multiple images were used per colony for the one year monitoring to ensure complete colony coverage because of increased colony complexity (Figs. S1B and S1C). Only colonies that survived the entire year were included in the growth and productivity analysis. Each colony was traced by three different researchers and the average TLE was used for analysis, when variation between TLE measurements was greater than 15% colonies were re-analyzed by all researchers. Annual productivity was estimated from the sum of length of tissue/coral produced over one year divided by the initial sum of length of tissue/coral per colony ((TLE_final_–TLE_initial_)/TLE_initial_) (Forrester et al., 2011; Lirman et al., 2014) and growth was estimated by TLE_final_–TLE_initial_.

Colony survival, productivity, growth, partial mortality, and prevalence of conditions were compared among size classes, attachment techniques, genotypes, and sites. Genotype 2 was excluded from this analysis because of a low number of replicates (Table S1). Data for each analysis were tested for normality using the Shapiro–Wilk test, normality assumptions were not met for survival, partial mortality, and prevalence of conditions data and therefore non-parametric tests were performed. Attachment success was evaluated using Kaplan–Meier survival analysis with log rank tests (Therneau, 2015). In order to evaluate success of attachment technique, missing colonies were considered dead in the survival analysis, because although missing colonies may not have died, they did not successfully attach and their fate was unknown. Kruskal–Wallis tests by ranks were used to explore the prevalence of conditions and partial mortality between size classes, attachment techniques, genotypes, and sites. Post hoc multiple comparisons of mean ranks between groups with a Bonferroni adjustment were employed when significant differences between groups were found. The Bonferroni correction was calculated by $p = 2 * \left( {1 - \Pr \left( {Z - z'} \right)} \right) * k * \left( {k - 1} \right)$, where *k* is the total number of groups in the comparison (Statistica 13.0©, Tibco, Palo Alto, CA, USA). One-way analysis of variance were used to assess the differences in colony productivity and growth (log(*x* + 100) transformed data) between colony size, attachment technique, genotype, and site. Post hoc comparisons between groups were performed using Tukey’s HSD tests. All analyses were performed using Statistica 13.0©.

### Density

**Figure 3:**
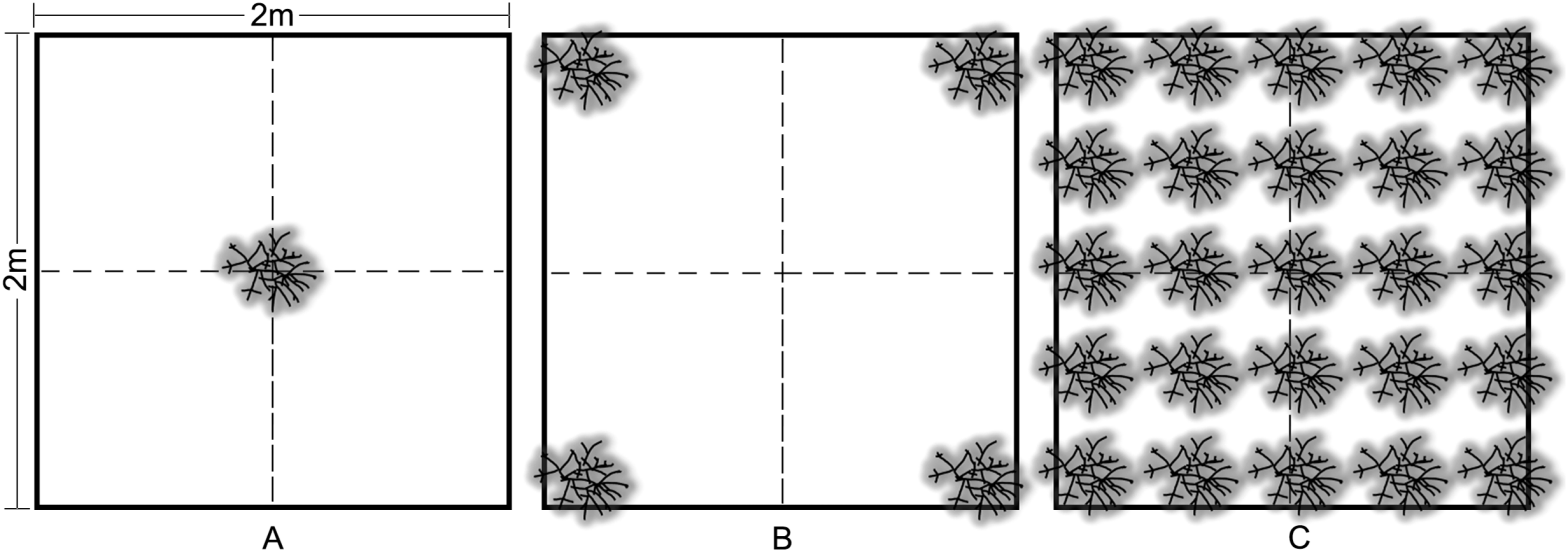
*Acropora cervicornis* outplant colony arrangement in 2 × 2 m density treatments: (A) low (1 coral/4 m^2^), (B) medium (4 corals/4 m^2^), and (C) high (25 corals/4 m^2^). Colonies were approximately 30 cm TLE and outplanted using a nail, cable tie, and epoxy.

Outplanting occurred in May 2013 at four sites on the nearshore ridge complex of Broward County, Florida at depths of 3–6 m. Colonies were outplanted to 4 m^2^ plots in three density treatments: (1) low—one colony, (2) medium—four colonies (2 m spacing), and (3) high—25 colonies (50 cm spacing) (Fig. 3). Three replicate treatments were installed at each site and arranged using a random block design with a minimum of 15 m between treatments. Wild *A. cervicornis* colonies within 5 m of each treatment were relocated within the site in order to avoid interference with the treatments. Outplant colonies of approximately 30 cm TLE from 11 genotypes were attached to the substrate using a masonry nail, cable tie and two-part epoxy. Multiple genotypes (randomized across all treatments and sites) were used to control for the effect of genotype and in turn represent a natural population, therefore genotype was not used as a factor in the analyses for this experiment.

Individual colony survival, partial mortality, and condition data were collected quarterly for two years, following the methods outlined above for the size and attachment technique experiment. These data were used to calculate plot survival, colony partial mortality, and prevalence of conditions. Data were divided into zero to one year post-outplanting and one to two years post-outplanting to evaluate the effects that increasing colony size and cover of the treatments had on colony survival, partial mortality, and condition. Plot survival and conditions were compared among treatments, between years, and between sites. Survival of each plot was calculated at the end of one and two years by dividing the number of colonies alive by the total number of colonies at the start of each year. Kruskal–Wallis tests by ranks were used to explore plot survival, the prevalence of conditions, and partial mortality between treatments and years. Post hoc multiple comparisons of mean ranks between groups with a Bonferroni adjustment (see above) were employed when significant differences between groups were found.

All nursery and outplanting related research was conducted pursuant to Florida Fish and Wildlife Conservation Commission issued Special Activity Licenses: SAL-10-1086A-SCRP; SAL-11-1086A-SCRP; SAL-13-1086-SCRP; SAL-13-1086C-SCRP; SAL-14-1086-SCRP; SAL-14-1086A-SCRP.

## Results

### Size and attachment technique

**Figure 4:**
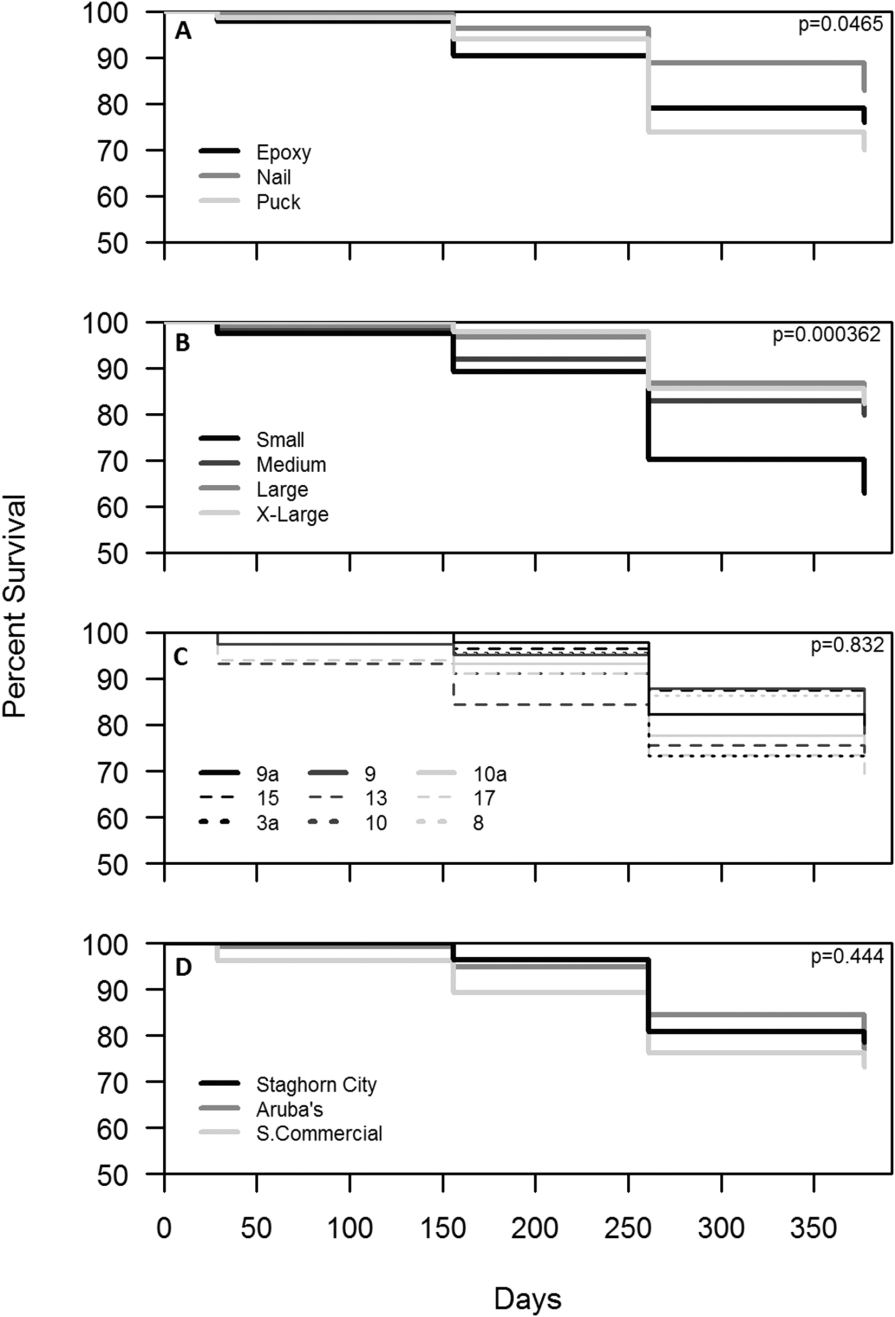
Survival analysis (Kaplan–Meier *p* < 0.05) of outplanted *Acropora cervicornis* colonies after one year by attachment technique (A), size class (B), genotype (C), and site (D).

Survival for all treatments combined after one year was 77%. Colonies outplanted using a nail and of larger size classes had the highest survival (Figs. 4A and 4B). Colony survival differed significantly between attachment techniques (*Χ*^2^ = 6.47; *df* = 2; *p* < 0.05), size classes (*Χ*^2^ = 18.52; *df* = 3; *p* < 0.05), within the small size class between techniques (*Χ*^2^ = 11.74; *df* = 2; *p* < 0.05), and within epoxy and puck techniques between size classes (*Χ*^2^ = 19.74; *df* = 3; *p* < 0.05 for all comparisons). Genotype and site did not have a significant effect on the survival of outplanted colonies (Figs. 4C and 4D; *Χ*^2^ = 4.87; *df* = 8; *p* > 0.05; *Χ*^2^ = 1.35; *df* = 2; *p* = 0.51). A majority of the mortality was observed during the eight-month monitoring event (Fig. 4). Seventeen percent of the colonies became dislodged and were recorded as missing. All size classes, techniques, sites, and genotypes (except Genotype 15) had missing colonies. However, the number of missing colonies was only significantly different among attachment techniques and size classes (Kruskal–Wallis; *H* = 9.65 and 10.41; *p* < 0.05).

**Figure 5:**
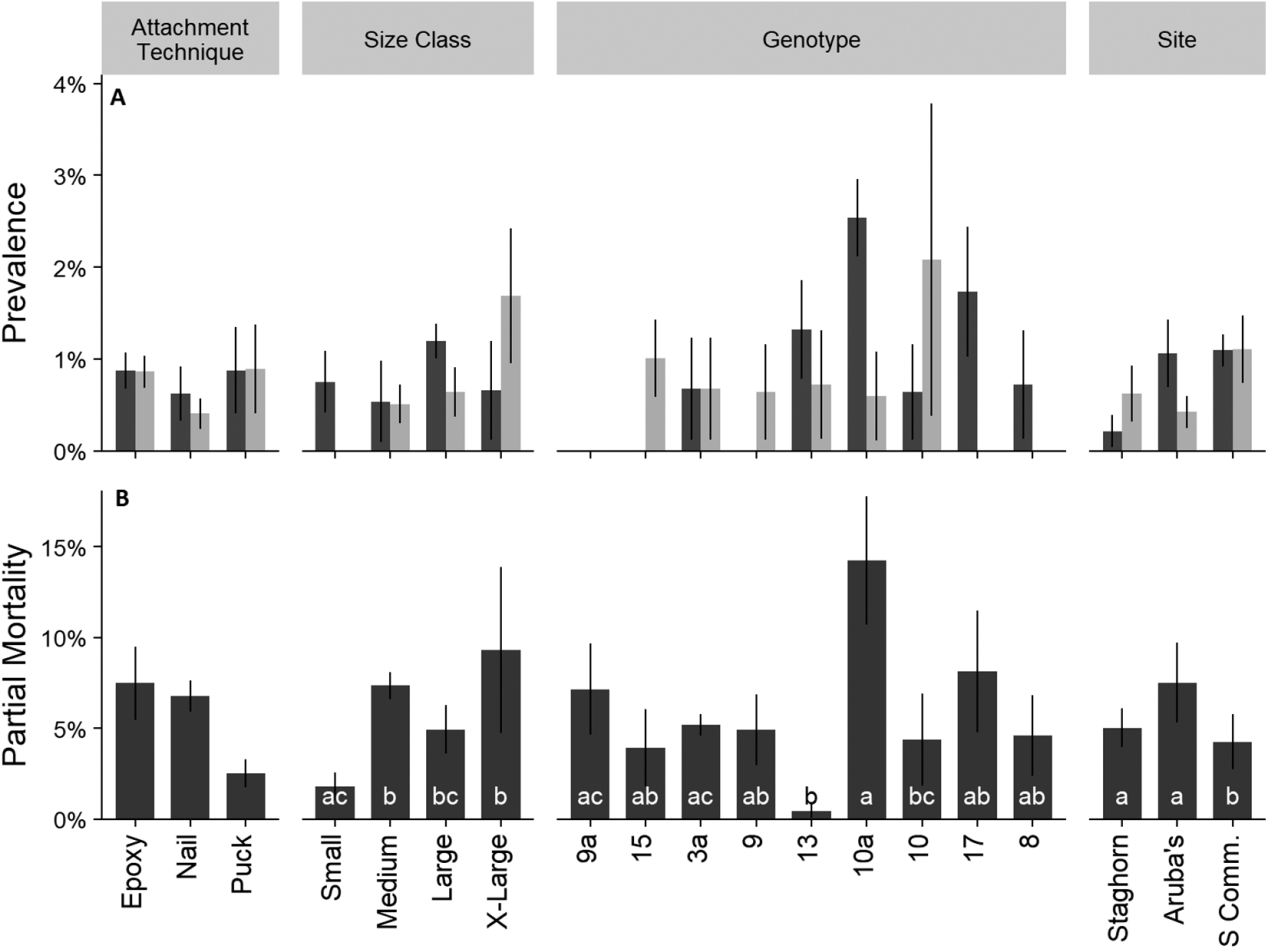
Cumulative prevalence of conditions on outplanted *Acropora cervicornis* colonies after one year by attachment technique, size class, genotype, and site. Panel (A)—mean cumulative prevalence of disease (dark gray), and predation (light gray). Panel (B)—mean partial mortality. Different letters within groups indicate significant differences between factors *p* < 0.05, Kruskal–Wallis multiple comparisons. Where there were no significant differences letters were removed for clarity. Error bars indicate ±1 SE.

Mean percent partial mortality, not including colonies that died, was 5.7 ± 0.93 SE% and was attributed to disease, predation, sediment burial, or unknown causes. Total prevalence of disease (rapid tissue loss and white band disease) and predation, by *Coralliophila abbreviata* (corallivorous snail), were lower than 1.5% during each monitoring event (Fig. 5A). Predation by fireworms (*Hermodice carunculata*) was not observed. Mean partial mortality was significantly different amongst size classes, genotypes, and sites (Fig. 5B; Kruskal–Wallis; *H* = 15.22, 14.33, and 9.13; *p* < 0.05 for all comparisons). Treatment did not have a significant effect on the prevalence of disease or predation (Fig. 5; Kruskal–Wallis; *p* > 0.05).

**Figure 6:**
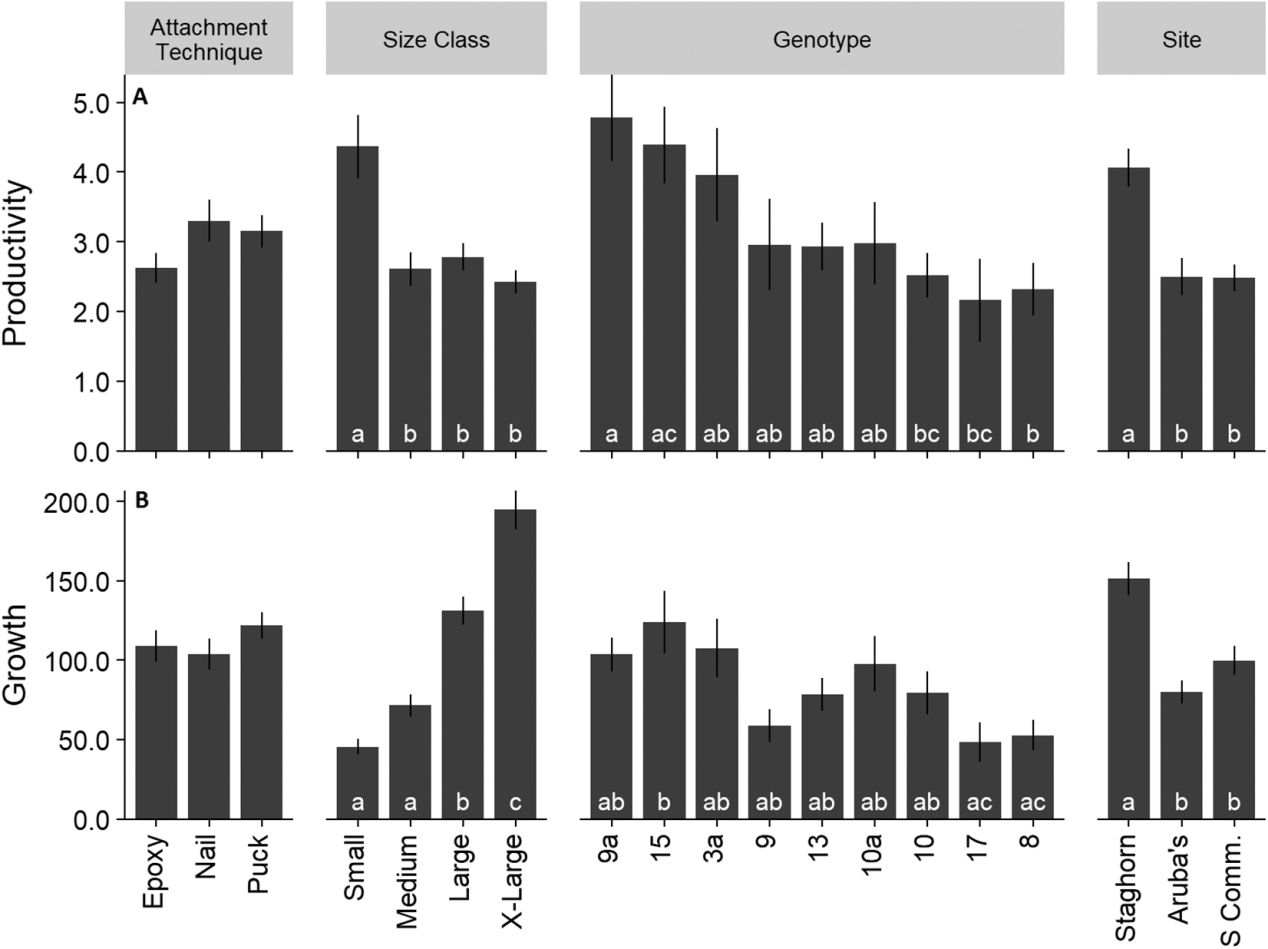
*Acropora cervicornis* outplant colony productivity (A) (cm/cm of initial tissue) and growth (B). Letters on bars indicate significant differences within groups *p* < 0.05, Tukey HSD. Where there were no significant differences letters were removed for clarity. Error bars indicate ±1 SE.

**Figure 7:**
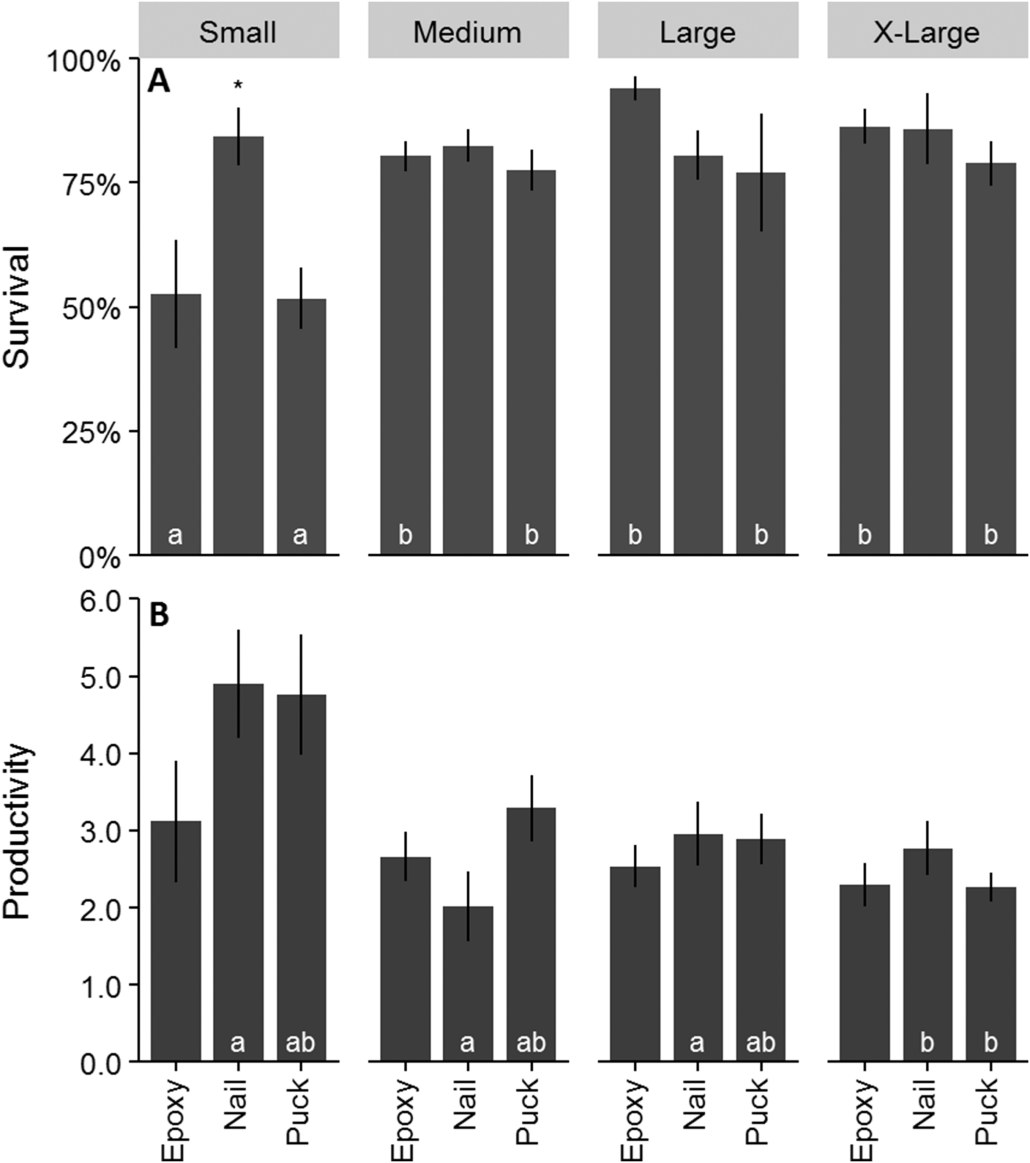
Survival and productivity of outplanted *Acropora cervicornis* colonies after one year. These data show the combined effect of colony size and attachment technique. Mean survival per colony (A) and mean productivity per colony (B). Letters on bars indicate significant differences within attachment techniques across size classes and within size class differences are indicated by an asterisks *p* < 0.05, Kruskal–Wallis and log rank tests for survival and Tukey HSD for productivity. Where there were no significant differences letters were removed for added clarity. Error bars indicate ±1 SE.

Mean colony productivity (sum of all branch lengths) was 3.03 ± 1.5 SE cm/cm initial tissue length, with 32,533 cm of new coral produced from the 12,643 cm coral initially outplanted. Mean growth rate was 111.15 ± 5.4 cm/year. Productivity was similar across all three attachment techniques (Fig. 6A; *F* = 1.92; *df* = 2; *p* > 0.05). Small colonies had significantly higher mean productivity (4.37 ± 4.5 cm/cm initial tissue length) than any other size class (Fig. 6A; *F* = 9.71; *df* = 3; *p* < 0.05). Productivity varied significantly among genotypes ranging from 2.2 to 4.5 cm/cm of initial tissue length (Fig. 6A; *F* = 3.68; *df* = 8; *p* < 0.05). Colonies outplanted at Staghorn City had a significantly higher productivity than the other two sites (*F* = 13.714; *df* = 2; *p* < 0.05). There were no significant differences in productivity within a size class between attachment techniques (*F* = 1.49; *df* = 6; *p* = 0.18). Colonies attached with epoxy or pucks had significantly higher survival with larger colonies (Fig. 7A; Tukey HSD *p* < 0.05 for both comparisons). Small colonies attached with nails and pucks had significantly higher mean productivity than medium and x-large colonies, respectively (Fig. 7B; Tukey HSD *p* < 0.05 for both comparisons). Colony growth or the amount of coral produced per fragment increased significantly with colony size (Fig. 6B; *F* = 7.45; *df* = 8; *p* < 0.05) and between genotypes and sites (Fig. 6B).

### Density

**Figure 8:**
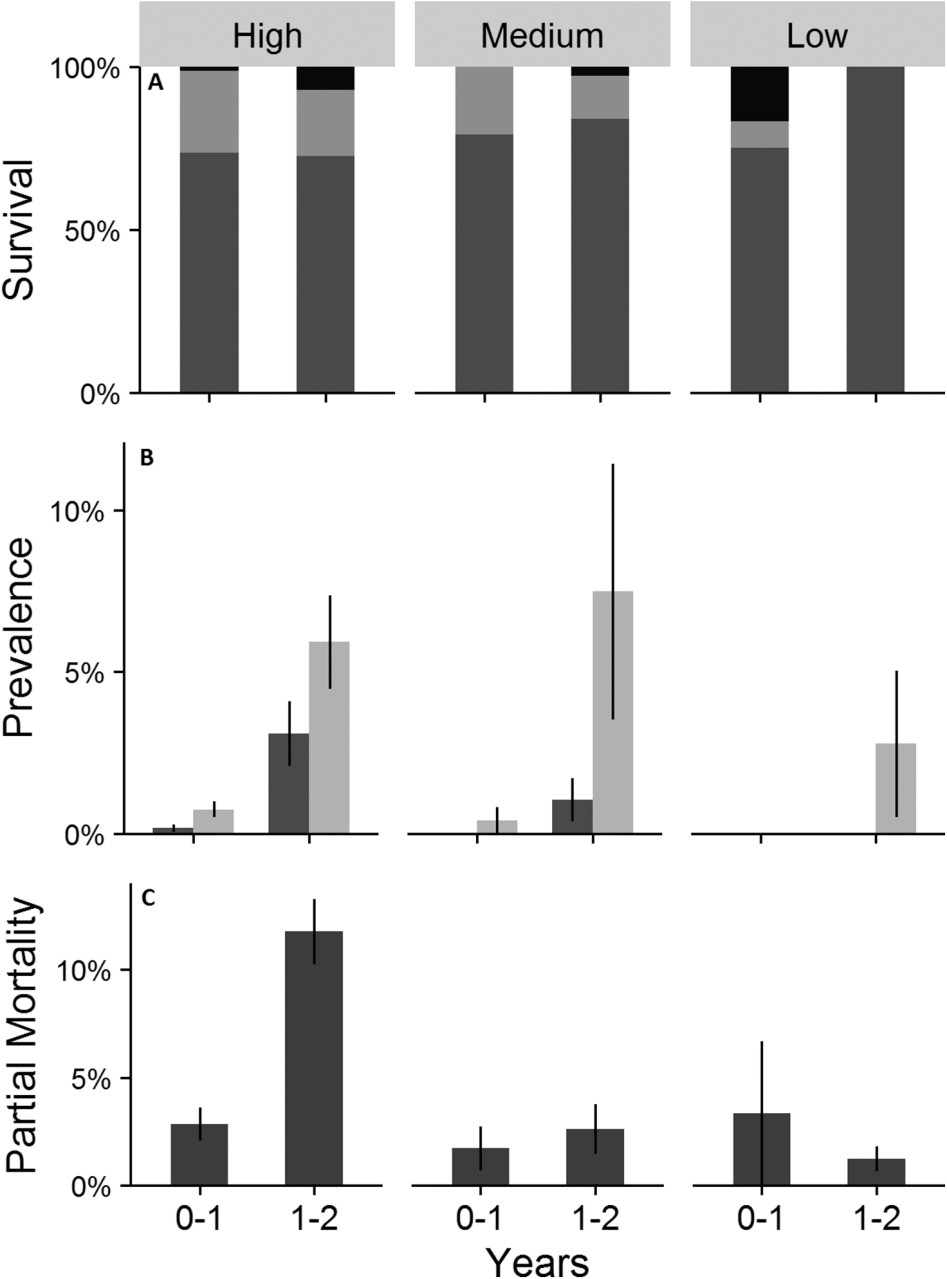
Survival (A), cumulative prevalence of conditions (B), and partial mortality (C) on outplanted *Acropora cervicornis* colonies. In (A) survival dark gray is alive, light gray is missing and black is dead. In (B) prevalence of disease is dark gray and prevalence of predation is light gray. Data are separated by 1 (0–1) and 2 years (1–2) for colonies outplanted in three densities: High (25 colonies/4 m^2^), Medium (4 colonies/4 m^2^), and Low (1 colonies/4 m^2^). A table indicating significant differences is found in the Table S2. Error bars indicate ±1 SE.

Survival between treatments was similar after one year (Fig. 8A; Table S2; Kruskal–Wallis; *H* = 4.76; *p* > 0.05), but significantly different after two years (Kruskal–Wallis; *H* = 15.96; *p* < 0.05). Low density treatments had the highest survival (Fig. 8A). Mean number of colonies missing was significantly higher in the high density treatments than the medium and low densities (Table S2; Kruskal–Wallis; *H* = 16.48; *p* < 0.05).

Predation by *H. carunculata* and *C. abbreviata* and disease (rapid tissue loss and white band disease) were the most commonly recorded conditions across two years (Fig. 8B). Prevalence of disease and predation were significantly higher in the high density treatments during the second year than the first year (Fig. 8B; Table S2; Friedman test; *Χ*^2^ = 5.44 and 8.33; *p* < 0.05). The only condition reported in the low density treatments was predation during year 2, which was significantly less than observed in the high density treatments in both years (Kruskal–Wallis; *H* = 12.13 and 6.75; *p* < 0.05). Disease was never recorded in the low density treatment and was significantly less than the high density treatment during year 2 (Fig. 8; Kruskal–Wallis; *H* = 10.58; *p* < 0.05).

Mean partial colony mortality increased significantly from the first to the second year for high density treatments (Fig. 8C; Table S2; Friedman test; *Χ*^2^ = 5.33; *p* < 0.05). Colony partial mortality was significantly different between treatments within both years (Fig. 8C; Table S2; Kruskal–Wallis; *H* = 9.06 and 15.99; *p* < 0.05). During the second year partial mortality of colonies in high density treatments was significantly higher than the medium and low density treatments (Fig. 8; Table S2; multiple comparisons; *p* < 0.001).

## Discussion

Our results suggest that outplanted colonies should be at least 15 cm TLE, spaced 1–2 m apart and attached using a nail and cable tie. Outplant efforts spread across multiple sites with a variety of genotypes will also increase the overall success of a restoration program. While there were a few genotypes that performed better (no disease and faster productivity and growth) this was only based on one year of data and could change between sites and years. The techniques used here maximize survival, ecological impact (creating habitat faster), the potential for cross-fertilization, and minimize the prevalence of disease.

Small colonies had higher productivity, but result in a lower ecological impact due to their lower growth rates, survival, morphologic simplicity (one to two secondary branches), and sexual immaturity compared to colonies in the larger size classes. Differences in productivity and survival between size classes may be attributed to changes in energy allocation, in addition larger colonies are able to overcome adverse conditions, such as sedimentation, disease, algae interaction, and predation (Loya, 1976; Sato, 1985; Forsman, Rinkevich & Hunter, 2006). As corals age and grow, reaching the size capable of sexual maturity, their energy allocation changes (Sebens, 1982; Meesters & Bak, 1995; Okubo, Motokawa & Omori, 2007). Corals in the small size class were not yet of the reported size of being sexually mature (Soong & Lang, 1992) and consequently all their energy may have been allocated to growth and regeneration, whereas the three other size classes are of a size capable of producing oocytes could be the cause of decreased productivity (Okubo, Taniguchi & Motokawa, 2005). Productivity trends of our outplanted colonies was similar to that previously described for *A. cervicornis* outplant and nursery colonies (Lirman et al., 2014) as well for Pacific corals (Loya, 1976; Yap et al., 1998). Productivity of small colonies during this experiment was similar to those reported by Lirman et al. (2014) for outplants in Florida, but our medium colonies were more similar to the outplant colonies in the Dominican Republic.

Differences in productivity and prevalence of conditions were seen amongst genotypes being raised in similar environments, reflecting what others have found (Osinga et al., 2011; Bowden-Kerby & Carne, 2012; Griffin et al., 2012; Lirman et al., 2014; Drury, Manzello & Lirman, 2017). However, the observed variability in prevalence of disease amongst genotypes does not necessarily indicate genotypic resistance. For example, two genotypes (15 and 17) used in both experiments revealed variable results; depending on site and year, survival ranged from 40% to 100% and prevalence of disease, predation, and bleaching ranged from 0% to 3.5%, 0% to 2.3%, and 1.2% to 4.4%, respectively within one genotype. These results support that caution should be used when selecting “best performing” genotypes for restoration, as evidence suggests that in addition to survival and prevalence of conditions, growth, productivity, and thermal resilience may vary between region, site, and years (Tunnicliffe, 1981; Harriott, 1998; Lirman et al., 2014; Miller et al., 2014; Drury, Manzello & Lirman, 2017). As many unknown factors can influence initial outplanting success (e.g., unpredictable storm events, temperature anomalies, regional disease outbreaks), restoration efforts should diversify outplant arrays across multiple sites, using a variety of genotypes. If genotype is not taken into consideration in restoration projects or if they are lumped together conditions maybe masked or exaggerated. In addition, maintaining genotypic diversity within restoration programs is imperative for successful sexual reproduction. Slower growing genotypes will not contribute as quickly to sexual reproduction if outplanted as small colonies, as sexual maturity of *A. cervicornis* has been linked to colony size (Soong & Lang, 1992). Therefore, it may be beneficial for restoration programs to initially outplant colonies of or close to reproductive size to increase the likelihood of cross-fertilization.

In 2015, a Recovery Plan for Elkhorn and Staghorn Corals was published by the United States National Marine Fisheries Service outlining objectives necessary to reach the ultimate goal of delisting these species as threatened under the United States Endangered Species Act (National Marine Fisheries Service, 2015). Under the first objective (“Ensure Population Viability”), a staghorn coral abundance criteria was defined as: thickets (≥0.5 m diameter colonies at a density of 1/m^2^ or live staghorn coral cover of ∼25%) present on 5% of the consolidated reef habitat in the fore reef zone throughout the species range and maintained for 20 years (National Marine Fisheries Service, 2015). As restoration programs grow, practitioners are moving toward massive high density outplanting projects focusing on meeting this criterion. However, our results indicate that outplanting at this density (1 colony/m^2^) or higher, while it may create habitat complexity more quickly, decreases the survival of the colonies and increases the prevalence of disease and predation over time. Ladd et al. (2016) reported this same trend although colony health in their study didn’t significantly deteriorate until a density of 3 colonies/m^2^. Although this tradeoff seems counter intuitive as historical populations of *A. cervicornis* were recorded in high densities, recall that disease killed these high density populations leaving behind remnant individuals which have continued to exist as isolated colonies and are now the material for the recovery of this species. While there are still high density populations in existence today they are few and far between and have the propensity to die or experience a great reduction in live tissue within years (E. Goergen, 2008–2017, personal observation). While the etiology and process of disease-induced mortality is still being explored, we have demonstrated that disease may spread more quickly and have a bigger impact on outplanted colonies which are relocated within very close proximity (<0.5 m) to each other supporting the theory that for this species disease can be spread by contact, vectors such as *C. abbreviata* or *H. carunculata* (Williams & Miller, 2005; Miller & Williams, 2007; Vollmer & Kline, 2008; Kline & Vollmer, 2011) or currents and that predators may be drawn to higher density populations for more protection or increased abundance of prey (Berkle, 2004). This pattern of increased disease and predation was not unique to outplanted colonies and was observed on wild populations surrounding the outplant sites; affecting areas of congregated colonies or patches more commonly than isolated colonies (E. Goergen, 2008–2017, personal observation). Furthermore, the frequency of disease reported on Arabian Gulf and Australian Reefs was greatest at high coral cover sites that had a high frequency of sea surface temperature anomalies (Riegl, 2002; Bruno et al., 2007) so as the oceans continue to warm this trend could be even further exacerbated in high density populations.

The biggest cause of colony loss during this study was colonies which were displaced from their place of outplanting. While colony attachment by nail and cable tie reduced the number of colonies going missing by at least 10%, when compared to the other methods, fate tracking of colonies after two years was difficult (Bruckner & Bruckner, 2001; Bruckner, Bruckner & Hill, 2009; Garrison & Ward, 2012; Hollarsmith, Griffin & Moore, 2012; Mercado-Molina, Ruiz-Diaz & Sabat, 2014). High frequency of missing colonies may not be indicative of outplanting failure, but is simply a characteristic of *A. cervicornis* life history, as missing colonies were often found attached to the reef meters away from the location they were outplanted. In addition, the rate of wild colony dislodgement (50% loss after two years (E. Goergen, 2010–2012, unpublished data)) and control colonies (Garrison & Ward, 2008) was similar to what we report for outplanted colonies. The impact of outplanting corals can be seen beyond the outplanting areas and is missed by tracking each individual where it was outplanted, especially for ephemeral coral species that are known to propagate easily through fragmentation. To quantify the site impact of coral restoration through population enhancement, prior to outplanting, wild colony abundance assessments should be made and repeated periodically following outplanting. These periodic assessments would determine the natural propagation rates of this species and aid in defining the long-term success of population enhancement of an ephemeral species.

The methods presented here were successful in terms of survival, over 70% after one year, increasing local abundance of *A. cervicornis*, and creating habitat complexity. These were just three attachment techniques that were available to our outplanting program and used by other outplanting programs within Florida and the Greater Caribbean. At the time of this project, the cost of materials to outplant approximately 100 corals using epoxy was $60 USD, nail and cable tie $15 USD, and puck $190 USD. Not only did the puck technique cost more in supplies, it was also the most time consuming in terms of creating and deploying the pucks at the outplant site. It took two people about 3 h to make 100 cement pucks, which had to cure at least 24 h. They also must be attached to the substrate, at a minimum the amount of time it takes for the epoxy (or cement) to set, before outplanting to ensure attachment. If this is not done the weight and drag of the coral may dislodge the puck from the substrate before it has the time to attach. Outplanting colonies with epoxy took about 2–3 min each and depended on the size and how many attachment points were needed. Pounding in nails depended on substrate type, but was a quick process taking about 1–2 min to pound in the nail and attach the colony. In one day, with an experienced crew of five divers, colonies were collected from the nursery (1 h dive) and outplanted to one site (2–2 h dives). This process would be accelerated if experimental design was not a factor and sites were closer. There are many other costs involved that will influence the total cost of an outplanting program and were not included here because they are very dependent on diver experience, nursery to outplant site distance, outplanting design, site condition, and availability of resources and supplies. However, from our experience herein the added cost and time of making and deploying pucks for outplanting is not countered with greater success or colony performance and therefore should be used as a last resort. There is not one single solution to successful outplanting, but we present a number of factors that will influence and increase the success of an outplanting program especially as restoration efforts continue to scale up.

## Supplemental Information

10.7717/peerj.4433/supp-1 Supplemental Information 1 Size and technique outplant data.

10.7717/peerj.4433/supp-2 Supplemental Information 2 Supplemental material.

## Additional Information and Declarations

### Field Study Permissions

The following information was supplied relating to field study approvals (i.e., approving body and any reference numbers): Research was conducted pursuant to Florida Fish and Wildlife Conservation Commission issued Special Activity Licenses: SAL-10-1086A-SCRP; SAL-11-1086A-SCRP; SAL-13-1086-SCRP; SAL-13-1086C-SCRP; SAL-14-1086-SCRP; SAL-14-1086A-SCRP.

### Data Availability

The following information was supplied regarding data availability: The raw data has been provided as Supplemental Dataset Files.
