# Supplementary material for: Outplanting technique, host genotype, and site affect the initial success of outplanted Acropora cervicornis
Source: PeerJ. 2018 Feb 28;6:e4433. doi: 10.7717/peerj.4433 (PMC5834935; doi:10.7717/peerj.4433)
Supplement: Supplemental Information 2 [file peerj-06-4433-s002.docx]

**Outplanting technique, host genotype, and site affect the initial success of outplanted Acropora cervicornis**

Elizabeth A. Goergen^1^ and David S. Gilliam^1^

Nova Southeastern University Halmos College of Natural Sciences and Oceanography 8000 N. Ocean Drive, Dania Beach, FL, USA

Email: [goergen@nova.edu](mailto:goergen@nova.edu)

**Supplemental Material:**


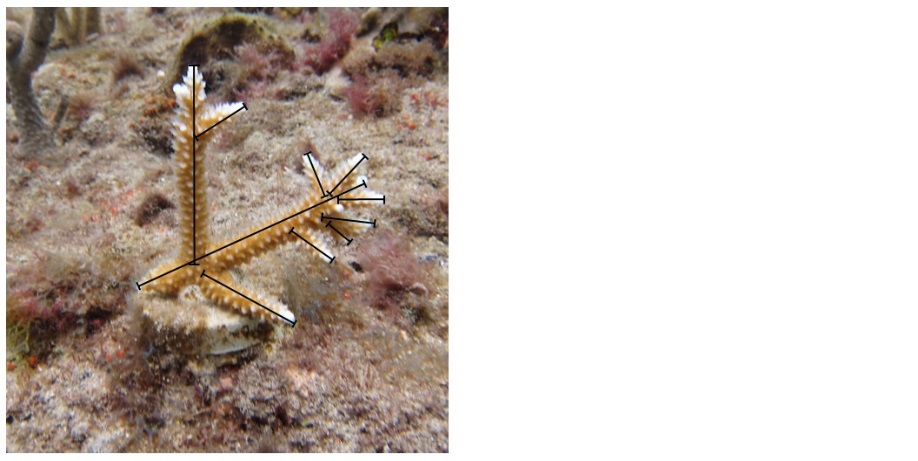

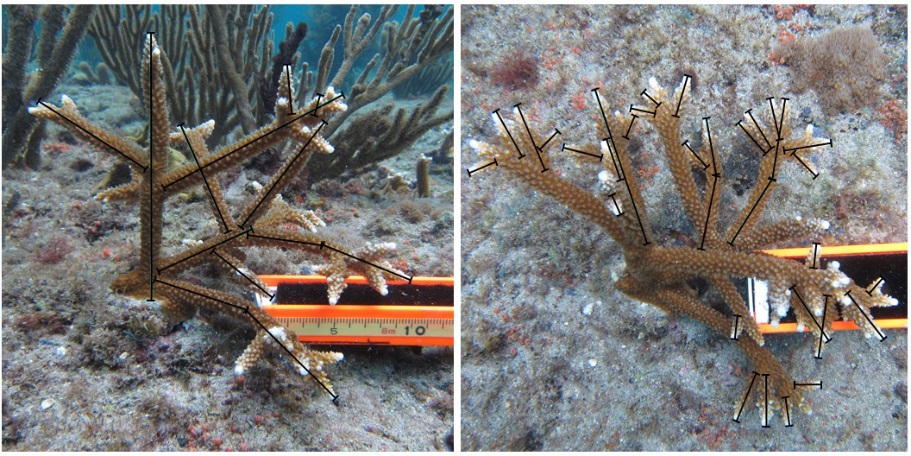


A B C

Figure S1. Outplanted *Acropora cervicornis* colony images were analyzed using Coral Point Count with Excel extensions 4.1© to determine change in colony size. A change in total linear extension (TLE) was estimated by tracing the length of every colony branch (denoted by the black lines on the images) initially (A) and at one year post-outplanting (B and C).

Table S1. Number of outplanted *Acropora cervicornis* colonies by genotype, attachment technique, and size class.

| **Size Class** | **Technique** | **2** | **8** | **9** | | **10** | | **13** | | **15** | | **17** | **3a** | **9a** | **10a** | **Total** |
| --- | --- | --- | --- | --- | --- | --- | --- | --- | --- | --- | --- | --- | --- | --- | --- | --- |
| **Small** | **Epoxy** |  | 5 | 4 | | 4 | | 5 | | 5 | | 5 | 3 | 5 | 4 | 40 |
|  | **Nail** |  | 2 | 7 | | 5 | | 9 | | 5 | | 6 | 3 | 6 | 1 | 44 |
|  | **Puck** |  | 4 | 4 | | 5 | | 5 | | 5 | | 4 | 5 | 2 | 3 | 37 |
| **Medium** | **Epoxy** |  | 3 | 7 | | 4 | | 5 | | 1 | | 5 | 2 | 5 | 2 | 34 |
|  | **Nail** |  | 9 | 4 | | 2 | | 1 | | 2 | | 4 | 4 | 4 | 5 | 35 |
|  | **Puck** |  | 3 | 3 | | 4 | | 4 | | 3 | | 6 |  | 5 | 2 | 30 |
| **Large** | **Epoxy** |  | 3 | 3 | | 4 | | 2 | | 5 | | 1 | 3 | 4 | 5 | 30 |
|  | **Nail** |  |  | 3 | | 4 | | 4 | | 4 | |  | 6 | 4 | 4 | 29 |
|  | **Puck** |  | 5 | 5 | | 2 | | 4 | | 3 | |  | 2 | 4 | 6 | 31 |
| **X-Large** | **Epoxy** | 2 | 4 |  | | 3 | | 3 | | 6 | | 1 | 7 | 1 | 4 | 31 |
|  | **Nail** | 1 | 3 |  | | 4 | | 1 | | 9 | | 1 | 2 | 1 | 5 | 27 |
|  | **Puck** | 2 | 3 | 1 | | 4 | | 2 | | 8 | | 1 | 8 | 4 | 4 | 37 |
| **Total by Genotype** | | 5 | 44 | | 41 | | 45 | | 45 | | 56 | 34 | 45 | 45 | 45 | 405 |

Table S2. Significant differences in colony survival, missing, disease, predation and partial mortality. ns= no significant difference, H, M or L indicates which density is significantly different from the row density for each condition; Post-hoc multiple comparisons of mean ranks (*p*<0.05). If a significant difference was found between years within a condition the relationship is shaded (Friedman Test (*p*<0.05)).

|  | **Survival** | | **Missing** | | **Disease** | | **Predation** | | **Partial Mortality** | |
| --- | --- | --- | --- | --- | --- | --- | --- | --- | --- | --- |
|  | 0-1 | 1-2 | 0-1 | 1-2 | 0-1 | 1-2 | 0-1 | 1-2 | 0-1 | 1-2 |
| **High** | ns | L | L | M,L | **ns** | **L** | **ns** | **L** | **L** | **M,L** |
| **Medium** | ns | L | ns | H | ns | ns | ns | ns | ns | H |
| **Low** | ns | H,M | H | H | ns | H | ns | H | H | H |
